# Long-term treatment of Cushing’s disease with pasireotide: 5-year results from an open-label extension study of a Phase III trial

**DOI:** 10.1007/s12020-017-1316-3

**Published:** 2017-06-09

**Authors:** S. Petersenn, L. R. Salgado, J. Schopohl, L. Portocarrero-Ortiz, G. Arnaldi, A. Lacroix, C. Scaroni, S. Ravichandran, A. Kandra, B. M. K. Biller

**Affiliations:** 1 ENDOC Center for Endocrine Tumors, Hamburg, Germany; 2 Division of General Internal Medicine, Hospital das Clínicas, University of São Paulo Medical School, São Paulo, Brazil; 3 Medizinische Klinik IV, University of Munich, Munich, Germany; 4 Department of Neuroendocrinology, National Institute of Neurology and Neurosurgery, Mexico City, Mexico; 5 Division of Endocrinology, Polytechnic University of Marche Region, Ancona, Italy; 6 Division of Endocrinology, Centre hospitalier de l’Université de Montréal, Montreal, Canada; 7 Endocrinology Unit, Department of Medicine, Padova University Hospital, Padova, Italy; 8 Novartis Pharmaceuticals Corporation, East Hanover, NJ USA; 9 Novartis Pharma AG, Basel, Switzerland; 10 Neuroendocrine Clinical Center, Massachusetts General Hospital, Boston, MA USA

**Keywords:** Cushing’s disease, Pasireotide, Clinical trial, Long-term, Phase III

## Abstract

**Background:**

Treating hypercortisolism in patients with Cushing’s disease after failed surgery often requires chronic medication, underlining the need for therapies with favourable long-term efficacy and safety profiles.

**Methods:**

In a randomised, double-blind study, 162 adult patients with persistent/recurrent or de novo Cushing’s disease received pasireotide. Patients with mean urinary free cortisol at/below the upper limit of normal or clinical benefit at month 12 could continue receiving pasireotide during an open-ended, open-label phase, the outcomes of which are described herein.

**Results:**

Sixteen patients received 5 years of pasireotide treatment. Among these, median (95% confidence interval) percentage change from baseline in mean urinary free cortisol was −82.6% (−89.0, −41.9) and −81.8% (−89.8, −67.4) at months 12 and 60. Eleven patients had mean urinary free cortisol ≤ upper limit of normal at month 60. Improvements in clinical signs were sustained during long-term treatment. The safety profile of pasireotide at 5 years was similar to that reported after 12 months. Fifteen of 16 patients experienced a hyperglycaemia-related adverse event; glycated haemoglobin levels were stable between months 6 and 60. Adverse events related to hyperglycaemia, bradycardia, gallbladder/biliary tract, and liver safety were most likely to first occur by month 6; adverse event severity did not tend to worsen over time.

**Conclusions:**

This represents the longest prospective trial of a medical therapy for Cushing’s disease to date. A subset of patients treated with pasireotide maintained biochemical and clinical improvements for 5 years, with no new safety signals emerging. These data support the use of pasireotide as an effective long-term therapy for some patients with Cushing’s disease.

## Introduction

Cushing’s disease is a rare and often severe disorder of chronic hypercortisolism caused by hypersecretion of adrenocorticotropic hormone (ACTH) from a corticotroph adenoma [1]. Untreated, Cushing’s disease is associated with significant morbidity (including metabolic, cardiovascular and psychiatric disorders), impaired quality of life and increased mortality [2–4].

Transsphenoidal resection of the causative pituitary adenoma is first-line therapy for most patients with Cushing’s disease [5]. However, many patients require pharmacological intervention in order to reduce excess levels of cortisol. With an increasing number of therapeutic agents available, clinicians face the challenging task of selecting the most appropriate therapy for each patient. Tailoring treatment to meet the individual patient’s characteristics along with a consideration of the benefit:risk profiles of the various medical therapies is recommended [5]. Given that Cushing’s disease often requires chronic medical treatment, medications with favourable long-term efficacy and safety profiles are required to meet the needs of patients and their clinicians.

Twice-daily pasireotide (Signifor)—a multireceptor-targeted somatostatin analogue [6]—has been approved for the treatment of adult patients with Cushing’s disease with persistent or recurrent hypercortisolism after surgery or for whom surgery is not an option [5]. Earlier reports from a large Phase III study (NCT00434148) showed that pasireotide reduced urinary free cortisol (UFC) levels and provided clinical benefit for up to 24 months in patients with Cushing’s disease [7–9]. The safety profile of pasireotide was similar to that of other somatostatin analogues, except for a higher frequency and degree of hyperglycaemia [7–9]. To date, published data on the efficacy and safety of pasireotide beyond 24 months of treatment have been limited to individual case reports or small patient series [10–14]. While these reports have shown disease control to be sustained for several years in individual patients, prospective data in a cohort of patients with Cushing’s disease have not been available. Long-term data not only establish durability of efficacy and other clinical benefits, but also potentially identify important safety and tolerability signals, which may only emerge over an extended duration of treatment.

Here, we report the safety and efficacy of pasireotide in patients (*n* = 16) who received 5 years of treatment during an extension to the Phase III study.

## Methods

### Patients and study design

Adults with de novo, persistent or recurrent Cushing’s disease were enroled in the Phase III study (Clinicaltrials.gov, NCT00434148) as previously described [7]. Patients were randomly assigned to receive double-blind subcutaneous pasireotide 600 µg or 900 µg twice daily (bid). Patients with a mean urinary free cortisol (mUFC) level not exceeding the upper limit of the normal range (ULN) or who were considered by the investigator to be achieving clinical benefit from pasireotide at month 12 could continue receiving pasireotide in an open-label phase. If treatment benefit persisted and pasireotide was well tolerated, patients could continue to receive pasireotide until it became commercially available. Increase and decrease of dose was permitted during the open-label phase for efficacy and tolerability issues (permitted dose range, 150–1200 µg bid). The study was approved by an independent ethics committee, research ethics board, or institutional review board at each centre and complied with the Declaration of Helsinki, the Harmonised Tripartite Guideline for Good Clinical Practice from the International Conference on Harmonisation, and local laws. All patients provided written informed consent.

### Assessment of efficacy and clinical signs

Patients attended a scheduled assessment visit every 2 weeks up to day 90, then monthly up to month 12 and every 3 months thereafter. mUFC, serum cortisol and plasma ACTH levels, as well as clinical signs of Cushing’s disease—systolic blood pressure (SBP) and diastolic blood pressure (DBP), body weight, body mass index (BMI) and bone mineral density (BMD)—were assessed as described previously [7]. mUFC, serum cortisol and plasma ACTH levels were analysed by central laboratories (Eurofins Medinet BV, Breda, The Netherlands; CRL Medinet Inc., Lenexa, KS, USA; and Eurofins Technology Services [Suzhou] Co Ltd, Suzhou, China); see Supplementary Appendix for collection and assay details. Facial rubor, cutaneous striae, bruising, supraclavicular and dorsal fat pads, and muscle strength were assessed as described previously [9]. Severity of rubor, striae, bruising and fat pads was scored on a scale of 0 to 3 (0, no signs; 1, mild; 2, moderate; 3, severe). Tumour volume was assessed by magnetic resonance imaging (MRI) and evaluated by a central reader (BioClinica Inc, Lyon, France).

### Safety assessments

Safety was assessed from baseline to study end by monitoring for adverse events (AEs); severity of AEs was graded according to Common Terminology Criteria for Adverse Events (CTCAE) version 3.0 [15]. Haematological and blood biochemical measurements, including of fasting plasma glucose (FPG) and glycated haemoglobin (HbA_1c_) levels, were performed as previously described [7]. Concomitant medications (excluding drugs for the treatment of Cushing’s syndrome) were permitted at the discretion of the investigator; dose information for concomitant medications was not recorded.

### Statistical methods

Median (95% confidence interval [CI]) values for mUFC, serum cortisol, plasma ACTH, and clinical signs were calculated in patients who had evaluable measurements at the specific time point; for calculations of actual or percentage change, only those patients who had evaluable measurements at baseline and the later time points were included.

Efficacy data are presented at various time points up to month 60 for all patients who received at least one dose of pasireotide (overall population) and for those patients who reached month 60. Safety data are presented from baseline up to study end (defined as the time at which the last patient discontinued the study) for the overall population and for patients who reached month 60. Life-table estimates were used to calculate annualised rates of first-reported AEs related to bradycardia, hyperglycaemia, the gallbladder/biliary tract and the liver (AEs of special interest) during the following time intervals: 0–6 and >6–12 months, and >1–2, >2–3, >3–4, >4–5 and >5–6 years.

## Results

### Patients

Seventy-eight of the 162 patients who were randomised and treated completed 12 months of treatment, and 58 patients continued to receive pasireotide beyond month 12. Of these, 41, 19, and 16 patients were still receiving treatment at months 24, 48 and 60, respectively; the maximum duration of exposure to pasireotide was 76.6 months.

Baseline (month 0) characteristics for the overall population and for the 16 patients who reached month 60 are shown in Table 1.

**Table 1:** Summary of baseline characteristics for the overall population (*n* = 162) and the 16 patients who reached month 60

Characteristic	Overall population	Reached month 60
	*N* = 162	*N* = 16
Females, *n* (%)	126 (77.8)	14 (87.5)
Age		
Mean, years (range)	40.2 (18–71)	44 (24–67)
≥65 years, *n* (%)	5 (3.1)	1 (6.3)
Mean time since diagnosis, months (range)	54 (0–372)	63 (5–149)
Previous treatment, *n* (%)		
Surgery	128 (79.0)	14 (87.5)
Medication	78 (48.1)	9 (56.3)
Pituitary irradiation	7 (4.3)	3 (18.8)
Cushing’s disease status, *n* (%)		
De novo	27 (16.7)	1 (6.3)
Persistent/recurrent	135 (83.3)	15 (93.8)
Tumour volume		
Baseline measurement, *n*	75	6
Median, cm^3^ (range)	0.23 (0.02‒22.83)	0.08 (0.02, 0.20)
mUFC		
Baseline measurement, *n*	153	16
Median, nmol/24 h (range)	564.5 (195.0–22,943.8)	488.3 (219.5–1642.5)
mUFC, *n* (%)		
≤2 × ULN	26 (16.0)	3 (18.8)
>2–5 × ULN	66 (40.7)	7 (43.8)
>5–10 × ULN	41 (25.3)	5 (31.3)
>10 × ULN	20 (12.3)	1 (6.3)
Serum cortisol		
Baseline measurement, *n*	162	16
Median, nmol/l (range)	691 (291–1710)	668 (291–1189)
ACTH		
Baseline measurement, *n*	162	16
Median, pmol/l (range)	13 (0–109)	10 (3–25)

Baseline UFC was calculated if ≥3 samples were collected. Baseline tumour volume was calculated for patients with a measurable pituitary tumour on MRI. Normal ranges: UFC, 30–145 nmol/24 h; ACTH, 0–10 pmol/l

The most common reasons for patients discontinuing the study (≥5% of patients) between baseline and study end were unsatisfactory therapeutic effect (32.7%), AEs (22.2%), withdrawal of consent (18.5%) and administrative problems (9.9%). Reasons for patient discontinuation by treatment year are shown in Supplementary Fig. 1. Most of those patients who discontinued for reasons of unsatisfactory therapeutic effect or AEs did so during the first 12 months of treatment.

### Long-term efficacy

#### Urinary free cortisol

**Fig. 1:**
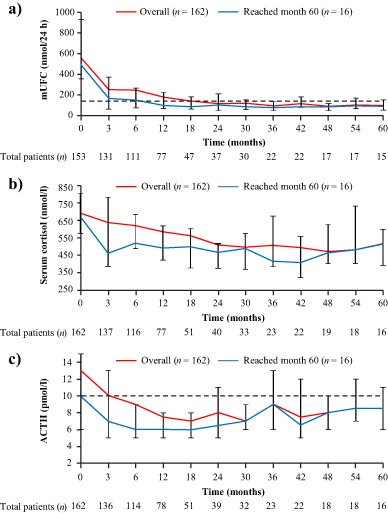
Median **a** mUFC, **b** serum cortisol and **c** ACTH levels from baseline up to month 60. Error bars show 95% distribution-free confidence limits for median values. Numbers of patients with evaluable measurements are shown beneath each time point for the overall population. *Dashed lines* represent ULN for UFC (145 nmol/24 h) and ACTH (10 pmol/l). Data for the overall population (*n* = 162) have previously been reported up to month 12 [7] and month 24 [8]

Overall, there was a sustained decrease in median mUFC levels from baseline up to month 60 (Fig. 1). In patients who reached month 60, decreases in median mUFC were seen within 3 months of treatment and were below ULN at month 12 through to month 60. In these patients, the median percentage change from baseline in mUFC was –82.6% (95% CI: –89.0, –41.9) at month 12 and –81.8% (95% CI: –89.8, –67.4) at month 60 (Fig. 1).

At month 60, 11/16 (68.8%) patients had controlled mUFC (mUFC ≤ ULN), 2/16 (12.5%) patients had partially controlled mUFC (mUFC > ULN but ≥50% decrease from core baseline), and 3/16 (18.8%) patients had uncontrolled mUFC. Of the 11 controlled patients at month 60, eight were previously controlled, one was partially controlled, and two were uncontrolled at month 12. Individual mUFC levels decreased gradually over time in the two patients who were controlled at month 60 but uncontrolled at month 12 (Supplementary Fig. 2a); neither of these patients had received prior pituitary irradiation. Two other patients with controlled mUFC at month 60 had undergone pituitary irradiation >10 years prior to study start; mUFC levels were controlled in both of these patients within 1 month of pasireotide treatment. One patient with uncontrolled mUFC at month 60 had also undergone prior pituitary irradiation.

Two patients who were controlled at month 12 were partially controlled at month 60 (Supplementary Fig. 2b). For one of these patients, mUFC was only marginally above ULN at month 60 (152 nmol/24 h; ULN, 145 nmol/24 h) and had returned below ULN at the next assessment (116 nmol/24 h [month 63]) without an increase in dose.

#### Serum cortisol and plasma ACTH

Decreases from baseline in median serum cortisol and plasma ACTH levels were apparent at month 3 and were sustained up to month 60 (Fig. 1). In patients who reached month 60, the median percentage change from baseline in serum cortisol was −28% (95% CI: −34.1, −6.4) at month 12 and –25% (95% CI: −41.6, 3.8) at month 60; the median percentage change from baseline in plasma ACTH at months 12 and 60 was −34.7% (95% CI: −45.5, 26.7) and −8.3% (95% CI: −33.3, 50.0), respectively (Fig. 1).

#### Clinical signs and tumour volume

**Fig. 2:**
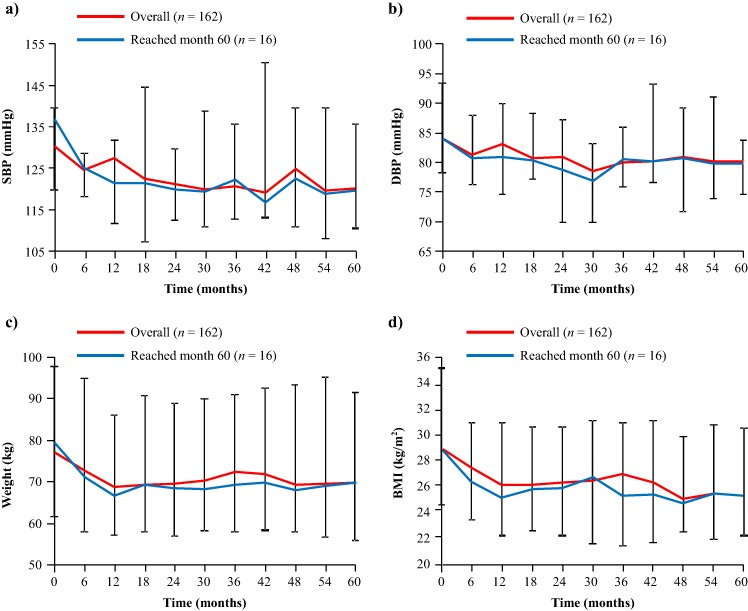
Median **a** SBP, **b** DBP, **c** weight, and **d** BMI from baseline up to month 60. Error bars show 95% distribution-free confidence limits for median values. Data for the overall population (*n* = 162) have previously been reported up to month 12 [7] and month 24 [8]

Clinically relevant decreases in SBP, DBP, weight, and BMI were observed within the first 6 months of pasireotide treatment (Fig. 2). These improvements were sustained up to month 60. Median changes from baseline to month 60 for the 16 patients who remained on pasireotide treatment were: SBP, −4.3 mmHg (95% CI: −17.3, 5.3); DBP, −1.7 mmHg (95% CI: −10.3, 3.3); weight, −6.2 kg (95% CI: −9.3, −1.8); and BMI, −2.3 kg/m^2^ (95% CI: −3.5, −0.6) (Fig. 2). Six of the 16 patients who reached month 60 were receiving antihypertensive drugs at study baseline. A further two patients initiated antihypertensive medication after starting pasireotide.

Most patients had the same or an improved severity score for facial rubor, cutaneous striae, bruising, and dorsal and supraclavicular fat pads at month 60 compared with baseline values. Muscle strength scores remained the same in most patients from baseline to month 60 (Supplementary Fig. 3). There were no significant changes seen from baseline to month 60 in median lean body mass (−2.1 kg [95% CI: −5.4, 0.6]), total body fat (−3.5 kg [95% CI: −10.0, 1.9]) and BMD: lumbar vertebrae, 0.0 mg/cm^3^ (95% CI: −0.1, 0.1); left proximal femur total hip, 0.0 mg/cm^3^ (95% CI: −0.1, 0.0); and left proximal femur neck, 0.0 mg/cm^3^ (95% CI: −0.1, 0.0). No significant changes were seen from baseline to month 60 in blood lipids: total cholesterol, −1.3 mmol/l (95% CI: −1.6, 0.0); low-density lipoprotein cholesterol, −0.7 mmol/l (95% CI: −1.0, 0.5); high-density lipoprotein cholesterol, −0.2 mmol/l (95% CI: −0.7, 0.1); and triglycerides, −0.3 mmol/l (95% CI: −0.6, 0.0).

Six of the 16 patients who reached month 60 had an evaluable tumour assessment at baseline. Median percentage change in tumour volume from baseline to last available assessment was –3.5% in those patients who reached month 60. Changes in tumour volume for individual patients who reached month 60 are shown in Supplementary Table 1.

### Long-term safety

Almost all patients who entered the core study (98.1%; 159/162) experienced ≥1 AE while receiving pasireotide (Table 2). The most common AEs (occurring in ≥30% of the overall population) from baseline up to study end were diarrhoea (58.6%), nausea (53.7%), hyperglycaemia (41.4%), cholelithiasis (32.7%), and headache (30.9%) (Table 2). A similar safety profile was seen for patients who reached month 60 (Table 2). When AE terms were grouped (e.g., all terms relating to elevations in blood glucose), 75.3% (122/162) of patients in the overall population and 93.8% (15/16) of patients who reached month 60 experienced at least one AE related to hyperglycaemia. Of the overall patient population and those who reached month 60: 37.7% (61/162) and 62.5% (10/16) experienced a gallbladder/biliary-related AE; 15.4% (25/162) and 25.0% (4/16) experienced a bradycardia-related AE; and 17.3% (28/162) and 18.8% (3/16) experienced a liver-safety-related AE, respectively.

**Table 2:** AEs (regardless of study drug relationship) occurring in ≥10% of the 162 patients who received pasireotide

Preferred term	Overall population (*N* = 162)		Patients reaching month 60 (*N* = 16)	
	Grades 3/4	All grades	Grades 3/4	All grades
	*n* (%)	*n* (%)	*n* (%)	*n* (%)
Diarrhoea	5 (3.1)	95 (58.6)	0	7 (43.8)
Nausea	4 (2.5)	87 (53.7)	2 (12.5)	11 (68.8)
Hyperglycaemia	21 (13.0)	67 (41.4)	2 (12.5)	9 (56.3)
Cholelithiasis	3 (1.9)	53 (32.7)	1 (6.3)	8 (50.0)
Headache	3 (1.9)	50 (30.9)	1 (6.3)	7 (43.8)
Abdominal pain	4 (2.5)	41 (25.3)	1 (6.3)	8 (50.0)
Diabetes mellitus	14 (8.6)	36 (22.2)	2 (12.5)	8 (50.0)
Fatigue	3 (1.9)	36 (22.2)	1 (6.3)	8 (50.0)
Nasopharyngitis	0	24 (14.8)	0	6 (37.5)
Alopecia	0	21 (13.0)	0	3 (18.8)
Hypercholesterolaemia	0	20 (12.3)	0	4 (25.0)
Abdominal pain upper	0	19 (11.7)	0	6 (37.5)
Asthenia	4 (2.5)	19 (11.7)	0	3 (18.8)
Dizziness	2 (1.2)	19 (11.7)	0	3 (18.8)
ALT increased	4 (2.5)	18 (11.1)	0	2 (12.5)
Gamma-glutamyltransferase increased	6 (3.7)	18 (11.1)	0	2 (12.5)
HbA_1c_ increased	1 (0.6)	18 (11.1)	0	1 (6.3)
Hypertension	0	18 (11.1)	0	2 (12.5)
Hypoglycaemia	3 (1.9)	18 (11.1)	0	3 (18.8)
Decreased appetite	0	17 (10.5)	0	2 (12.5)
Myalgia	1 (0.6)	17 (10.5)	1 (6.3)	8 (50.0)

*ALT* alanine aminotransferase

In total, 36 (22.2%) patients discontinued the study because of an AE. Of these discontinuations, 26 (72.2%) occurred before month 12; 29 (80.6%) and 35 (97.2%) occurred before months 24 and 36, respectively. The most common AEs that led to discontinuation (≥1% of patients) were diabetes mellitus (3.7%), diarrhoea (3.7%), hyperglycaemia (3.7%), elevated gamma-glutamyltransferase (3.1%), elevated alanine aminotransferase (1.2%), cholelithiasis (1.2%), fatigue (1.2%), and nausea (1.2%). No deaths were reported during the study.

**Fig. 3:**
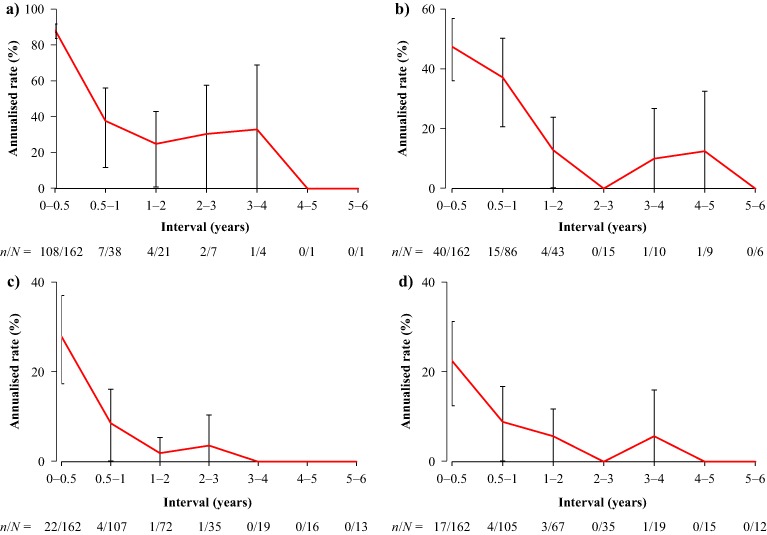
Annualised rates of first-reported AEs* related to **a** hyperglycaemia, **b** the gallbladder/biliary tract, **c** the liver, and **d** bradycardia. Data show annualised rates of first-reported AEs by time interval and 95% CI. During the first year of treatment, annualised rates were estimated for 0–6 and >6–12 months. The numbers of patients who experienced a first-reported AE (*n*) and the total number who were ‘at risk’ (*N*; ongoing at start of interval and had not already experienced the AE) are shown below each graph for each time interval. *Common AE terms were grouped, for example, all terms related to hyperglycaemia (e.g., elevated FPG/HbA_1c_ and diabetes mellitus) or liver safety

Annualised rates of first-reported AEs related to bradycardia (22.5%), hyperglycaemia (88.5%), the gallbladder/biliary tract (47.5%), and the liver (27.9%) were highest during the first 6 months of pasireotide treatment (Fig. 3). Steady decreases in annualised rates were seen after month 6 up to month 72.

In the overall population, the majority of AEs related to bradycardia, the gallbladder/biliary tract, hyperglycaemia, and the liver were CTCAE grade 1 or 2 at first occurrence. In most cases, the CTCAE grade of these AEs did not worsen at any time after first occurrence (Supplementary Table 2). For those patients who reached month 60, no increases in CTCAE grade were reported at any time after first occurrence for AEs related to bradycardia and liver safety. Increases in CTCAE grade were reported for 3/10 gallbladder/biliary-related AEs and 7/15 hyperglycaemia-related AEs; two and three AEs, respectively, worsened to CTCAE grade 3 (Table 3).

**Table 3:** CTCAE grade of AEs related to hyperglycaemia, the gallbladder/biliary tract, the liver, or bradycardia at first occurrence and worst value at any time after first occurrence for the 16 patients who reached month 60

Shaded boxes indicate higher CTCAE grades at worst reported value compared with first occurrence

#### Blood glucose changes

**Fig. 4:**
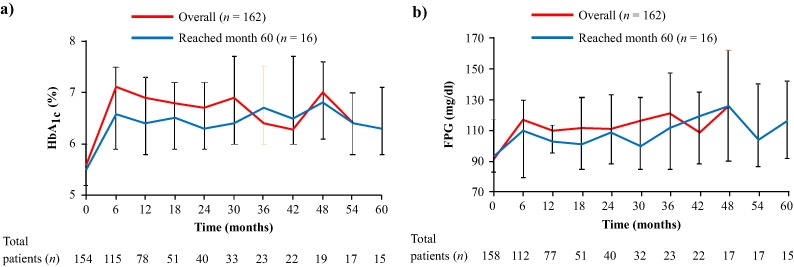
Median **a** HbA_1c_ and **b** FPG from baseline up to month 60. Error bars show 95% distribution-free confidence limits for median values. Numbers of patients with evaluable measurements are shown beneath each time point for the overall population. Concomitant treatment with antidiabetic medication was permitted at the discretion of the investigator. Data for the overall population (*n* = 162) have previously been reported up to month 12 [7] and month 24 [8]

Changes in median HbA_1c_ and FPG from baseline through to month 24 have previously been reported for the overall population [7, 8]; increases in median HbA_1c_ and FPG were seen by month 6, after which levels plateaued. In patients who reached month 60, median HbA_1c_ increased from 5.5% (95% CI: 5.2, 6.0) at baseline to 6.6% (95% CI: 5.9, 7.5) at month 6 and 6.3% (95% CI: 5.8, 7.2) at month 60. In these patients, median FPG increased from 93.7 mg/dl (95% CI: 82.9, 117.1) at baseline to 111.7 mg/dl (95% CI: 79.3, 129.7) and 117.1 mg/dl (95% CI: 91.9, 142.3) at months 6 and 60, respectively (Fig. 4).

Two of the 16 patients who reached month 60 were receiving antidiabetic medication at study baseline. A further 11 patients initiated one or more antidiabetic drugs after starting pasireotide; median time from study start to first antidiabetic medication was 52 weeks (range: 1‒226). For those patients who initiated antidiabetic therapy during the study, median HbA_1c_ levels increased from baseline (5.2% [95% CI: 5.1, 6.2]) to month 6 (6.7% [95% CI: 6.0, 7.7]) and then plateaued through to month 60 (6.4% [95% CI: 5.8, 7.3]).

## Discussion

In the longest-running prospective clinical trial of a medical therapy for Cushing’s disease to date, pasireotide treatment reduced median mUFC and improved disease-related clinical signs. These beneficial effects were sustained for up to 5 years in the subgroup of patients who remained on treatment. No new safety signals were identified during long-term treatment.

At the end of the study, 16 patients had completed ≥5 years of treatment. Median mUFC decreased within the first 3 months of treatment for these patients and remained suppressed up to month 60; median mUFC had decreased from baseline by 82% at month 60. The proportion of patients with controlled mUFC was similar at month 12 (*n* = 10/16; 62.5%) and month 60 (*n* = 11/16; 68.8%). Two patients showed a gradual response to pasireotide treatment; these patients had uncontrolled mUFC levels at month 12 but were subsequently controlled at month 60 (without history of pituitary irradiation). Conversely, two patients who were controlled at month 12 were only partially controlled at month 60. For one of these patients, mUFC was slightly elevated (<1.1 × ULN) at month 60 and had returned to within the normal range at the time of the next assessment (month 63) without dose adjustment. Median plasma ACTH and serum cortisol levels were also suppressed below baseline levels during the 5-year treatment period.

Compared with the enroled population, a similar proportion of patients who remained on pasireotide treatment for at least 5 years had baseline mUFC levels in the following ranges: ≤2 × ULN, >2−5 × ULN, and >5–10 × ULN. These findings demonstrate that pasireotide can provide long-term benefit to a subgroup of patients with varying degrees of hypercortisolaemia.

The reversal of clinical signs, symptoms and comorbidities represents an important goal for the treatment of Cushing’s disease [5]. Pasireotide treatment led to long-term improvements in SBP, DBP, weight, and BMI, which may mitigate the increased morbidity and mortality observed in patients with untreated Cushing’s disease [4]. Clinical improvements were sustained for up to 5 years. Other clinical signs of Cushing’s disease, which can have profound effects on patients’ quality of life [16] and include facial rubor, cutaneous striae, bruising, fat pads, and poor muscle strength, also improved during long-term pasireotide treatment. Although most patients with Cushing’s disease harbour a pituitary microadenoma, control of tumour volume is an important clinical goal [5]. Pasireotide treatment has been demonstrated to reduce tumour volume in patients with Cushing’s disease [17]. In this study, tumour volume remained controlled over 5 years of pasireotide treatment. Given its antiproliferative effect, pasireotide may be particularly beneficial in patients with a clinically relevant tumour mass and/or tumour progression [18].

The safety profile of pasireotide was similar at the end of the study to that previously reported after 12 and 24 months of treatment [7, 8]; AEs were generally mild or moderate. To assess potential changes in first-reported AE risk over time, interval-specific rates were calculated for bradycardia-related, gallbladder-related, hyperglycaemia-related and liver-related AEs, all of which were most likely to emerge during the first 6 months of pasireotide treatment, with steady decreases in first-reported AE rates after this time. As routine monitoring of patients was performed less frequently after month 12, it is possible that some AEs could be under-reported at later time points. As this analysis did not take into account repeat episodes or prolongation of AEs in individual patients, changes in the severity of these AEs over time were also assessed. The severity of AEs related to hyperglycaemia and the gallbladder/biliary tract did not increase after first occurrence in over half of patients on long-term pasireotide treatment, while the severity of AEs related to bradycardia and the liver did not tend to increase after the first reported event.

Hyperglycaemia is a well-characterised side effect of pasireotide treatment [19]. In this long-term study, hyperglycaemia was manageable with the use of antidiabetic medication, which was permitted during this study. For patients on long-term pasireotide treatment who initiated antidiabetic medication during the study (*n* = 11/16), median HbA_1c_ levels increased soon after initiation of pasireotide and plateaued at month 6 through to month 60. However, given the small number of patients who reached month 60, these results should be interpreted with caution. Blood glucose levels should be monitored in patients treated with pasireotide so that prompt action can be taken if levels rise. No specific recommendations for treatment of hyperglycaemia were included in the study protocol. As such, it was not possible to assess the effect of individual medications on glycaemic parameters. A clinical trial is ongoing to evaluate the efficacy of various antidiabetic therapies in controlling pasireotide-induced hyperglycaemia in patients with Cushing’s disease or acromegaly (clinicaltrials.gov identifier: NCT02060383).

A number of other medical therapies have demonstrated efficacy in patients with Cushing’s disease, which exert their effects by inhibiting cortisol production at the adrenal glands (adrenocortical steroidogenesis inhibitors), blocking cortisol action at peripheral tissues (glucocorticoid receptor antagonists), or inhibiting ACTH release from the pituitary adenoma (dopamine receptor agonists) [20]. It is necessary to understand the long-term efficacy—control of cortisol excess, tumour proliferation and clinical sequelae of the disease—and safety of each therapeutic option in order to provide best possible outcomes for patients [18, 21].

Prospective data, which are more robust and less subject to bias than retrospective data, on the long-term use of other medical therapies used to treat Cushing’s disease are limited. In a retrospective study of 200 patients with Cushing’s disease, 51 patients were treated with ketoconazole for at least 24 months (mean, 109 months). At last assessment, 64.7% of these patients achieved normalised UFC, while 23.5% had at least a 50% decrease [22]. Discontinuation of ketoconazole because of intolerance occurred in 20.5% of patients. In a retrospective series of 195 patients with Cushing’s syndrome, 38 patients were treated with metyrapone for a mean duration of 18 months; ≥64% achieved normalisation of cortisol secretion [23]. Metyrapone was generally well tolerated, and potential side effects of hypokalaemia, hypertension, and hirsutism were rarely reported. Unlike pasireotide, adrenal-directed agents cannot reduce ACTH levels or influence pituitary tumour volume. Finally, mifepristone is approved in the USA to control hyperglycaemia secondary to hypercortisolism in patients with Cushing’s syndrome who have type 2 diabetes mellitus/glucose intolerance and have failed/are not candidates for surgery [5]. Mifepristone has been shown to provide clinically significant weight loss in patients with Cushing’s syndrome over 2 years of treatment [24]. Patients receiving mifepristone should be closely monitored for increase in circulating ACTH and cortisol levels and pituitary tumour enlargement.

## Conclusions

The chronic nature of hypercortisolism associated with Cushing’s disease necessitates, in some patients, a durable and effective medical treatment with a favourable safety and tolerability profile. Patients who were benefiting from treatment with pasireotide at month 12 of the core study continued to derive long-term benefit, as evidenced in the current study. Reductions in mUFC and improvements in clinical signs of Cushing’s disease with pasireotide were maintained for up to 5 years, with a safety profile similar to that reported after 1 and 2 years of treatment. These data represent the longest duration of treatment in a prospective clinical trial of any medical therapy for Cushing’s disease and support pasireotide as a viable long-term therapeutic option for some patients.

## Electronic supplementary material

Supplementary Fig 1

## References

[1] Lacroix A, Feelders RA, Stratakis CA, Nieman LK (2015). Cushing’s syndrome. Lancet.

[2] Pivonello R, De Leo M, Cozzolino A, Colao A (2015). The treatment of Cushing’s disease. Endocr. Rev..

[3] Etxabe J, Vazquez JA (1994). Morbidity and mortality in Cushing’s disease: an epidemiological approach. Clin. Endocrinol..

[4] Feelders RA, Pulgar SJ, Kempel A, Pereira AM (2012). The burden of Cushing’s disease: clinical and health-related quality of life aspects. Eur. J. Endocrinol..

[5] Nieman LK, Biller BM, Findling JW (2015). Treatment of Cushing’s syndrome: an Endocrine Society clinical practice guideline. J. Clin. Endocrinol. Metab..

[6] Bruns C, Lewis I, Briner U, Meno-Tetang G, Weckbecker G (2002). SOM230: a novel somatostatin peptidomimetic with broad somatotropin release inhibiting factor (SRIF) receptor binding and a unique antisecretory profile. Eur. J. Endocrinol..

[7] Colao A, Petersenn S, Newell-Price J (2012). A 12-month Phase 3 study of pasireotide in Cushing’s disease. N. Engl. J. Med..

[8] Schopohl J, Gu F, Rubens R (2015). Pasireotide can induce sustained decreases in urinary cortisol and provide clinical benefit in patients with Cushing’s disease: results from an open-ended, open-label extension trial. Pituitary.

[9] Pivonello R, Petersenn S, Newell-Price J (2014). Pasireotide treatment significantly improves clinical signs and symptoms in patients with Cushing’s disease: results from a Phase III study. Clin. Endocrinol..

[10] Yedinak C, Brzana J, Fleseriu M (2013). Monitoring patient improvement parameters following pasireotide treatment in Cushing’s disease. Case Rep. Endocrinol..

[11] Trementino L, Cardinaletti M, Concettoni C (2015). Up-to 5-year efficacy of pasireotide in a patient with Cushing’s disease and pre-existing diabetes: literature review and clinical practice considerations. Pituitary.

[12] Mackenzie Feder J, Bourdeau I, Vallette S (2013). Pasireotide monotherapy in Cushing’s disease: a single-centre experience with 5-year extension of phase III Trial. Pituitary.

[13] Lu L, Duan L, Jin Z, Lu Z, Gu F (2013). Effective long-term treatment of Cushing’s disease with pasireotide—a case report. Endocr. Pract..

[14] Shimon I, Rot L, Inbar E (2012). Pituitary-directed medical therapy with pasireotide for a corticotroph macroadenoma: pituitary volume reduction and literature review. Pituitary..

[15] National Cancer Institute. Common Terminology Criteria for Adverse Events v3.0 (CTCAE). (2006). http://ctep.cancer.gov/protocolDevelopment/electronic_applications/docs/ctcaev3.pdf

[16] Badia X, Valassi E, Roset M, Webb SM (2014). Disease-specific quality of life evaluation and its determinants in Cushing’s syndrome: what have we learnt?. Pituitary.

[17] Simeoli C, Auriemma RS, Tortora F (2015). The treatment with pasireotide in Cushing’s disease: effects of long-term treatment on tumor mass in the experience of a single center. Endocrine.

[18] Petersenn S (2015). How to manage pasireotide, when using as medical treatment for Cushing’s disease. Endocrine.

[19] Henry RR, Ciaraldi TP, Armstrong D (2013). Hyperglycemia associated with pasireotide: results from a mechanistic study in healthy volunteers. J Clin. Endocrinol. Metab..

[20] Ferone D, Pivonello C, Vitale G (2014). Molecular basis of pharmacological therapy in Cushing’s disease. Endocrine.

[21] Colao A, Boscaro M, Ferone D, Casanueva FF (2014). Managing Cushing’s disease: the state of the art. Endocrine.

[22] Castinetti F, Guignat L, Giraud P (2014). Ketoconazole in Cushing’s disease: is it worth a try?. J. Clin. Endocrinol. Metab..

[23] Daniel E, Aylwin S, Mustafa O (2015). Effectiveness of metyrapone in treating Cushing’s syndrome: a retrospective multicenter study in 195 patients. J. Clin. Endocrinol. Metab..

[24] Fein HG, Vaughan TB, Kushner H, Cram D, Nguyen D (2015). Sustained weight loss in patients treated with mifepristone for Cushing’s syndrome: a follow-up analysis of the SEISMIC study and long-term extension. BMC Endocr. Disord..

