# Supplementary material for: Long-term treatment of Cushing’s disease with pasireotide: 5-year results from an open-label extension study of a Phase III trial
Source: Endocrine. 2017 Jun 9;57(1):156–65. doi: 10.1007/s12020-017-1316-3 (PMC5486525; doi:10.1007/s12020-017-1316-3)
Supplement: Supplementary file 1 — Supplementary Fig 1 [file 12020_2017_1316_MOESM1_ESM.pdf]

Long-term treatment of Cushing's disease with pasireotide: 5-year results from an open-label extension study of a Phase III trial

S Petersenn, LR Salgado, J Schopohl, L Portocarrero-Ortiz, G Arnaldi, A Lacroix,

C Scaroni, S Ravichandran, A Kandra, and BMK Biller

Supplementary appendix

Assay details

UFC values were determined by high-performance liquid chromatography (Alliance® 2795 High Throughput System, Waters Corp, Milford, MA, USA; normal UFC range: 30–145 nmol/24h [10.8–52.5 µg/24h]). All samples were analysed by central laboratories (Eurofins Medinet BV, Breda, The Netherlands; CRL Medinet Inc, Lenexa, KS, USA; and Eurofins Technology Services [Suzhou] Co Ltd, Suzhou, China). Blood samples were tested for serum cortisol (assay: ADVIA Centaur® CP Immunoassay System, Siemens Healthcare Diagnostics Inc, Tarrytown, NY, USA) and plasma ACTH (assay: Immulite® 2000 ACTH kit, DPC, Los Angeles, CA, USA). Saliva samples were tested for salivary cortisol (assay: cortisol ELISA RE52611, IBL-Hamburg GmbH, Germany).

Supplementary Fig. 1 Reasons for patient discontinuation by treatment year

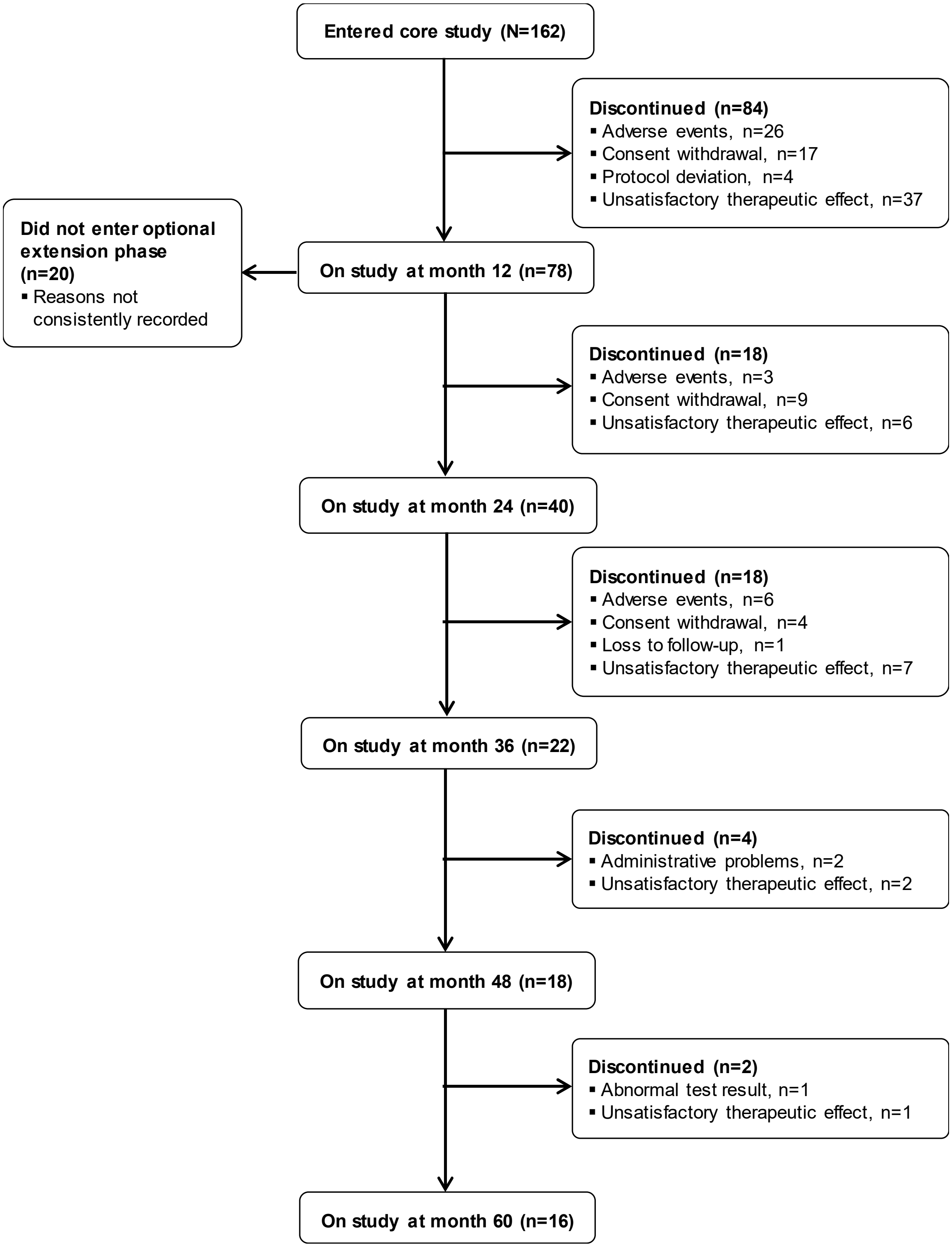

Supplementary Fig. 2 UFC levels from baseline up to month 60 in patients who were (a) uncontrolled at month 12 but controlled at month 60 (n=2) and (b) controlled at month 12 but partially controlled at month 60 (n=2)

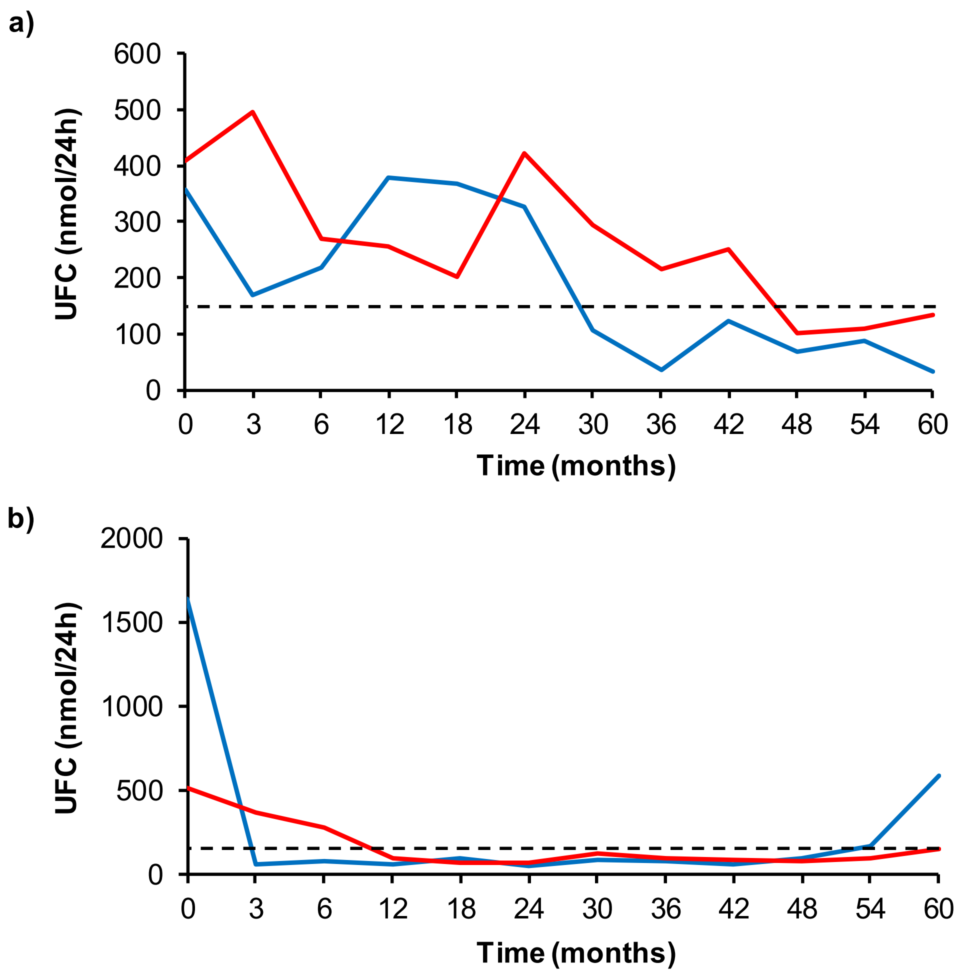

Dashed line represents ULN for UFC (145 nmol/24h). Data represent actual UFC values from baseline to month 60 for individual patients

Supplementary Fig. 3 Proportion of patients with a shift in signs of Cushing's disease at month 60 from baseline

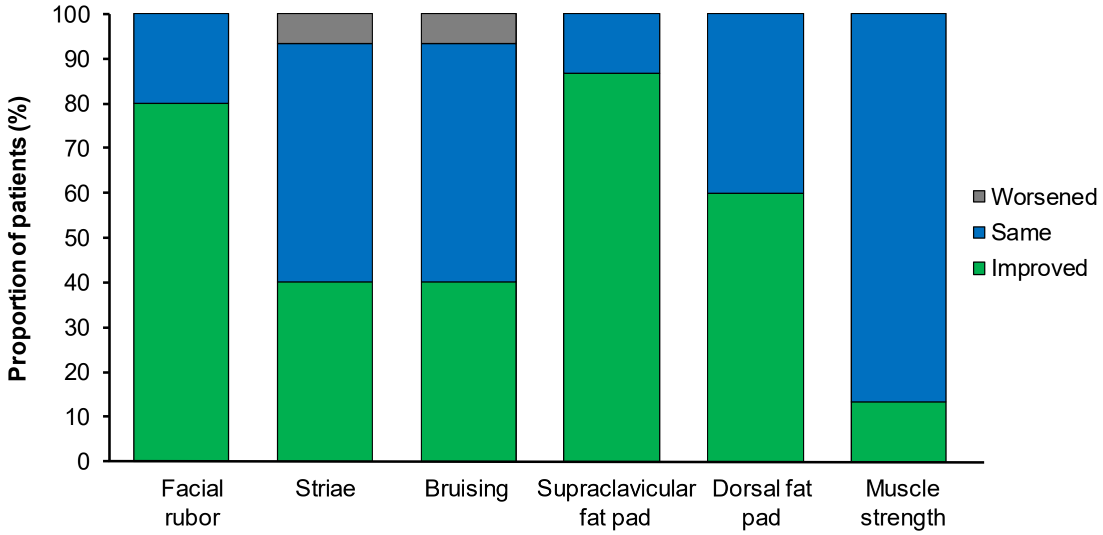

Facial rubor, striae, bruising and fat pads were assessed by a blinded reviewer using photographs (0=no signs, 1=mild, 2=moderate, 3=severe). Muscle strength was assessed according to the patient's ability to stand from a low seated position with arms extended (0=able to stand easily with arms extended, 1=able to stand after several efforts without using arms as assistance, 2=able to stand by using arms as assistance, 3=completely unable to stand). Fifteen patients had evaluable measurements at both month 60 and baseline

Supplementary Table 1 Changes in pituitary tumour volume in patients with evaluable measurements at baseline

Patient	Baseline, cm^3	Last available assessment, cm^3	Change to last post-baseline assessment, cm^3
1	0.20	0.03	−0.16
2	0.17	0.03	−0.14
3	0.10	0.36	+0.26
4	0.06	0.10	+0.04
5	0.04	0.09	+0.05
6	0.02	0.00	−0.02

Supplementary Table 2 CTCAE grade of AEs related to hyperglycaemia, the gallbladder/biliary tract, the liver, or bradycardia at first occurrence and worst value at any time after first occurrence (overall population)

First reported AE grade	n	AE reported once n (%)	Worst reported AE grade after first occurrence: Grade 1 n (%)	Grade 2 n (%)	Grade 3 n (%)	Grade 4 n (%)
**Bradycardia related**						
Grade 1	19	14 (73.7)	4 (21.1)	0	1 (5.3)	0
Grade 2	5	2 (40.0)	2 (40.0)	0	1 (20.0)	0
Grade 3	1	1 (100.0)	0	0	0	0
Grade 4	0	0	0	0	0	0
Total	*25*	*17*	*6*	*0*	*2*	*0*
**Gallbladder and biliary tract related**						
Grade 1	48	31 (64.6)	10 (20.8)	2 (4.2)	3 (6.3)	2 (4.2)
Grade 2	12	5 (41.7)	3 (25.0)	3 (25.0)	1 (8.3)	0
Grade 3	1	1 (100.0)	0	0	0	0
Grade 4	0	0	0	0	0	0
Total	*61*	*37*	*13*	*5*	*4*	*2*
**Hyperglycaemia related**						
Grade 1	56	20 (35.7)	12 (21.4)	15 (26.8)	9 (16.1)	0
Grade 2	44	20 (45.5)	5 (11.4)	8 (18.2)	11 (25.0)	0
Grade 3	20	8 (40.0)	1 (5.0)	7 (35.0)	4 (20.0)	0
Grade 4	2	0	0	1 (50.0)	1 (50.0)	0
Total	*122*	*48*	*18*	*31*	*25*	*0*
**Liver safety related**						
Grade 1	15	7 (46.7)	7 (46.7)	1 (6.7)	0	0
Grade 2	9	2 (22.2)	1 (11.1)	3 (33.3)	3 (33.3)	0
Grade 3	4	1 (25.0)	1 (25.0)	0	2 (50.0)	0
Grade 4	0	0	0	0	0	0

Total	*28*	*10*	*9*	*4*	*5*	*0*

Two bradycardia-, four gallbladder/biliary-, 20 hyperglycaemia-, and three liver-related AEs worsened to CTCAE grade 3 after the first event; two gallbladder/biliary-related AEs worsened to CTCAE grade 4 (Table 3). Worst reported CTCAE grades after first occurrence were lower than at the first reported event in a number of patients (bradycardia, n=2; gallbladder/biliary tract, n=3; hyperglycaemia, n=15; liver safety, n=2)
